# Ablative and Expansive Protocols for Bone Osteotomy in Rabbits

**DOI:** 10.3390/dj13030118

**Published:** 2025-03-07

**Authors:** Kazuhisa Kuwano, Luigi Canullo, Daniele Botticelli, Samuel Porfirio Xavier, Erick Ricardo Silva, Kaoru Kusano, Shunsuke Baba

**Affiliations:** 1 Department of Oral Implantology, School of Dentistry, Osaka Dental University, 8-1 Kuzuhahanazo-nocho, Hirakata 573-1121, Japan; kuwano_kazuhisa@yahoo.co.jp (K.K.); daniele.botticelli@gmail.com (D.B.); kusano-k@cc.osaka-dent.ac.jp (K.K.); baba-s@cc.osaka-dent.ac.jp (S.B.); 2 Department of Surgical Sciences (DISC), Largo Benzi, University of Genoa, 16100 Genova, Italy; 3 Department of Periodontology, University of Bern, 3010 Bern, Switzerland; 4 ARDEC Academy, 47923 Rimini, Italy; 5 Department of Oral and Maxillofacial Surgery and Periodontology, Faculty of Dentistry of Ribeirão Preto, University of São Paulo, Av. do Café-Subsetor Oeste-11 (N-11), Ribeirão Preto 14040-904, São Paulo, Brazil; spx@forp.usp.br (S.P.X.); erick.silva@usp.br (E.R.S.)

**Keywords:** implant preparation, drilling technique, bone response

## Abstract

**Background:** Cortical and marrow bone layer have different histomorphometric features. The traditional implant insertion technique provides for fixture stabilization through the cortical area. However, this approach has been found to result in an overstress of this bone layer, which may lead to resorption. Therefore, the aim of this study was to evaluate bone healing by applying two different implant site preparation protocols across various bone densities. **Materials and Methods:** One implant was placed in each femur and tibia of the rabbits (four implants per animal), using two distinct site preparation methods: drilling alone or drilling followed by osteotomes (funnel technique). Three regions around the implant were evaluated: cervical, marrow, and apical. The study included 12 rabbits, divided into two groups of 6 animals each, which were euthanized at 3 and 6 weeks, respectively (*n* = 6 per group). **Results:** In the cervical region of both femur and tibia, no marginal bone resorption could be detected. Similar BIC% (bone-to-implant contact percentages) were observed for funnel and drill sites after 3 weeks and 6 weeks of healing. Differences, though not statistically significant, ranged between 2.8% and 4.7%. However, higher BIC% values were observed in the femora compared to the tibia in both periods. **Conclusions:** No marginal bone loss was observed in both techniques. No statistically significant differences in bone resorption or bone-to-implant contact around the implant collar were observed when comparing two implant site preparation protocols across various bone densities. The use of osteotome did not influence the healing in the marrow region.

## 1. Introduction

Primary stability, also known as mechanical stability, results from the engagement of the implant fixture with the surrounding bone. Post-insertion, it is essential to minimize micromovements to maintain this stability [1,2,3,4].

Several factors influence primary stability and subsequent osseointegration, including bone volume, bone quality, surgical techniques for implant site preparation, and implant design. According to the classification by Lekholm and Zarb, bone can be categorized into four types (types 1, 2, 3, and 4) based on density [5]. From D1 to D4, there is a progressive decrease in the cortical bone component and an increase in the cancellous bone component. Consequently, D1 and D2 are considered high-density bone types, while D3 and D4 are classified as low-density bone types [5].

Implant site preparation can be performed using different methods, broadly categorized into ablative and expansive techniques. Drill osteotomy and ultrasonic osteotomy are examples of ablative procedures. Drill osteotomy is the conventional method for implant site preparation, involving the use of rotary instruments and burs of various shapes, geometries, and diameters to prepare the implant site in the mandibular or maxillary bone. The specific drilling sequence for implant osteotomy may vary depending on the implant manufacturer and bone phenotype.

Despite its widespread use, drill osteotomy has notable disadvantages, as documented in the literature. The use of drills can cause mechanical trauma and thermal injury to the bone, potentially leading to bone necrosis and compromising osseointegration. To prevent thermal damage, it is crucial to keep the temperature below 47 °C, which requires adequate irrigation during the procedure [6,7]. Multiple factors must be considered to avoid overheating during osteotomy, including rotational speed [8,9,10,11,12], drill geometry [8,13], frequency of drill reuse [14], applied pressure [15], intermittent drilling [16], bone hardness [14,17,18], and cooling methods [4,8,19,20,21,22]. Another limitation of traditional rotary instruments is the risk of damaging adjacent vessels, nerves, and soft tissues with the burs.

An alternative ablative method for implant bed preparation is ultrasonic osteotomy, first proposed by Horton in 1975 [23]. Ultrasonic devices are increasingly used across various dental fields and have been successfully applied in oral surgery for sinus floor augmentation and implant site preparation [24]. Sonic instruments are also utilized in several surgical applications [4,25,26].

Additionally, bone expansion can be performed using manual osteotomes [27] or a magnetic mallet [28]. This preparation technique was found particularly suitable for low-density bone (D4) and to fix vertical bony deficiency in the posterior maxillae [27].

Bone resorption is influenced by both bone type and the technique used for implant site preparation. One common method to enhance mechanical friction between the implant surface and peri-implant bone and to achieve better primary stability is the undersized drilling protocol, where the implant site is narrower than the implant diameter [29]. However, undersized osteotomy induces high lateral compression of the bone and results in a high insertion torque during implant placement. While the high insertion torque provides strong mechanical stability, it can also lead to bone resorption due to excessive mechanical stress [30].

Bone phenotype plays a crucial role in early bone adaptation, as cortical and cancellous bone respond differently to lateral compression. High lateral compression on cortical bone can lead to micro-fractures, necrosis, and bone resorption, whereas cancellous bone tends to remodel when subjected to compression [31,32]. Hypothetically, a significant mismatch between the prepared osteotomy and implant diameter, resulting in excessive lateral compression on cortical bone, could cause substantial bone resorption. However, in D4 bone types, where cortical bone is lacking, such resorption would not occur.

The literature presents a strong dichotomy between ablative and expansive techniques for implant site preparation. This study aims to bridge this gap by combining both approaches into a single, innovative method: the hybrid funnel technique. The objective is to shift the focus from “Ablative versus Expansive” to “Ablative plus Expansive”.

The hybrid funnel technique involves initial implant site preparation along the longitudinal axis using a drill, followed by the removal of cortical bone with a specially designed drill or a piezo-driven tip. Manual osteotomes are then used to compress the marrow bone [33]. Therefore, the aim of this study was to evaluate bone healing by applying two different implant site preparation protocols across various bone densities.

## 2. Materials and Methods

### 2.1. Ethical Statement

The protocol for the present randomized controlled animal study of within-subject comparison with blind assessment was approved by the Ethical Committee of the Faculty of Dentistry in Ribeirao Preto of the University of Sao Paulo (USP), Brazil (protocol 0057/2022R1 #2022.1.644.58.0). The ARRIVE guidelines were followed.

### 2.2. Study Design

In this preclinical study, a rabbit model was used, and implants were installed in the tibia and femur, bilaterally. Each rabbit received four implants: one in the proximal region of each tibia and one in the distal region of each femur, with the coronal margin of the implant positioned approximately at the level of the crests. Twelve rabbits were used in total, with six euthanized after 3 weeks and the remaining six after 6 weeks.

Rabbit tibia is a D2 bone type, with a cortical layer of about 1 mm width and no cancellous bone in the marrow space. Rabbit femur has a cortical layer of about 1.0 to 2.0 mm and cancellous bone morphology similar to D4 bone within the marrow space; The healing was studied after 3 and 6 weeks with early and late sacrifices.

Implants (NINA MultiNeO NH, Alpha-Bio Tec, Petah Tikva, Israel), measuring 10 mm in length and with diameters ranging from 3.2 to 3.75 mm, were utilized. These implants featured a surface with hydrophilic properties and a nanoscale roughness.

### 2.3. Experimental Groups

Tibia (D2 bone type)—The Hybrid Funnel Technique was used and NINA MultiNeO NH implant-surgical sites were prepared up to 10 mm in depth and 3.65 mm in diameter. A compressive mismatch of 0.1 mm between the implant fixture and the site preparation was yielded.

Tibia (D2 bone type)—Conventional drill osteotomy was used and a NINA MultiNeO NH implant-surgical site was prepared up to 10 mm in depth and 3.2 mm in diameter. A compressive mismatch of 0.55 mm between the implant fixture and the site preparation was yielded.

Femur (D4 bone type)—The Hybrid Funnel technique was used and a NINA MultiNeO NH implant-surgical site was prepared up to 10 mm in depth and 3.65 mm in diameter. A mismatch of 0.1 mm between the implant fixture and the site preparation was yielded.

Femur (D4 bone type)—Conventional drill osteotomy was used and a NINA MultiNeO NH implant-surgical site was prepared up to 10 mm in depth and 3.2 mm in diameter. A mismatch of 0.55 mm between the implant fixture and the site preparation was yielded.

### 2.4. Sample Size and Animals

In the absence of direct comparative data between the two procedures, the sample size was determined based on data from a previous study conducted on dogs, which examined the effects of different torque levels on bone compression [4]. This study identified a mean difference of 9.4, yielding an effect size of 2.57. Based on this effect size, with an α = 0.05, a power of 0.9, and a two-tailed test, the required sample size was calculated to be 5 pairs of animals to effectively reject the null hypothesis of no difference (G*Power 3.1.9.4). Consequently, 6 female New Zealand white rabbits, each weighing 3.5–4.0 kg and aged approximately 5–6 months, were used per group.

### 2.5. Randomization and Allocation Concealment

The randomization was performed by one author (S.P.X.) not involved in the surgery using randomization.com (last accessed on 19 December 2022). Two subjects were randomized in 6 blocks; the first six used it for a 3-week period and the second six for a 6-week period. The same randomization was used for both the femur and tibia. The allocation treatments were sealed in opaque envelopes and opened after the use of the 2–3 mm step drill in both the femur and tibia. The allocation indicated which technique to apply: funnel or conventional drill technique. All histological slides were coded to ensure that the assessors (E.R.S.) remained blinded to the assigned treatments.

### 2.6. Anesthetic Procedures

The anesthetic procedure was started using acepromazine I.M. (1.0 mg/kg; Acepran, Vetnil, Louveira, São Paulo, Brazil), followed by xylazine I.M. (3.0 mg/kg; Laboratórios Calier S/A, Barcelona, Spain), and ketamine I.M. (50.0 mg/kg; União Química Farmacêuti-ca Nacional S/A, Embuguaçú, São Paulo, Brazil). A single dose of oxytetracycline was used (0.2 mL/kg; Biovet; Vargem Grande Paulista, São Paulo, Brazil) for antibiotic therapy. After shaving and disinfection of the area with 1% polyvinylpyrrolidone iodine solution (Riodeíne Tintura, Rioquímica, São José do Rio Preto, São Paulo, Brazil), local anesthesia was injected (2% mepivacaine and 1:100,000 noradrenaline; Mepinor, Nova DFL, Rio de Janeiro, Brazil).

### 2.7. Surgical Procedure

**Figure 1:**
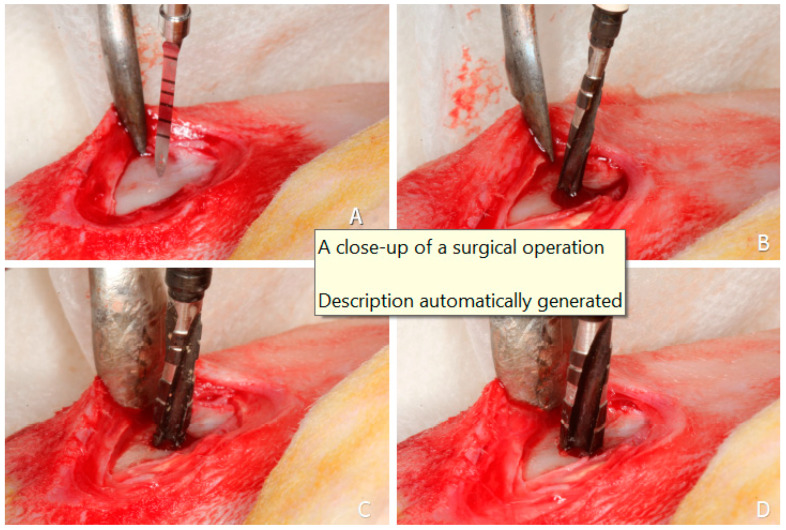
Clinical procedures. (**A**) lanceolate drill to mark the position; (**B**) 2.0 mm; (**C**) 2–3 mm step drill; (**D**) 3.65 mm used on the cortical layer.

**Figure 2:**
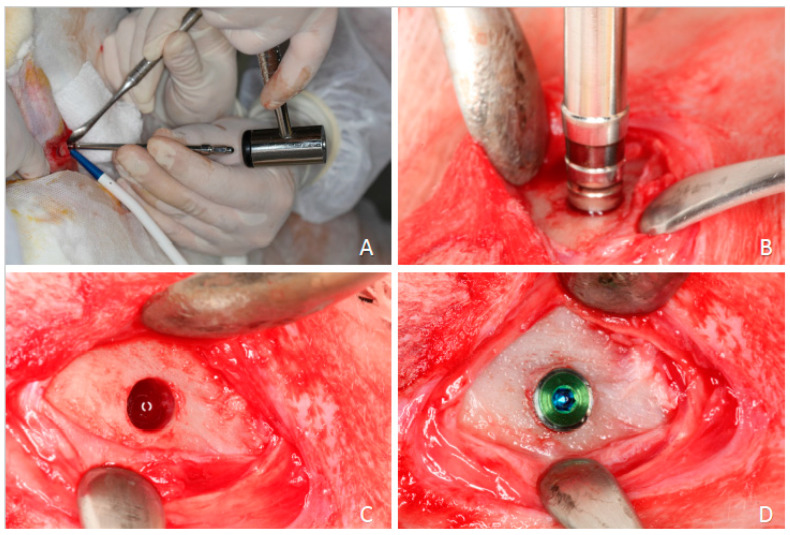
Clinical procedures. (**A**,**B**) specially designed osteotome to prepare the osteotomy; (**C**) osteotomy; (**D**) implant in position.

The surgeries were carried out by an experienced surgeon (V.F.B.; see acknowledgments). A 2.5 to 3.0 cm linear incision was performed over the skin in the proximal segment of the tibia and femur bilaterally, and the skin and periosteum were reflected. At this moment, the allocation of treatments was revealed to the surgeon. Two experimental sites were, respectively, identified: one in the tibial diaphysis and the other in the femoral diaphysis. Two different implant site preparations were prepared:

(1) Hybrid Funnel Technique: In D2 and D4 bones, the surgical site was prepared up to 10 mm in depth and 3.2 mm in diameter with a drilling bur. At the end of this stage, a 3.65 mm drill was used for the first 3 mm and a conical osteotome with the same shape of the implant body (3.75 mm in diameter at the implant collar) was adopted for osseodensification. The following drilling sequence was adopted:

- lanceolate drill (Figure 1A);
- 2.0 mm drill at implant length (Figure 1B);
- 2–3 mm step drill (Figure 1C);
- 3.65 mm drill removing the cortical bone (Figure 1D);
- specially designed osteotome with the implant body diameter for cancellous bone (Figure 2A,B);
- An implant was installed into the osteotomy with the coronal margin at the level of the cortical layer (Figure 2C,D).

(2) Conventional drill osteotomy: In D2 and D4 bones, the surgical site was prepared up to 10 mm in depth and 3.2 mm in diameter. The drilling sequence was performed as follows:

- 2.0 mm drill at implant length;
- 2–3 mm step drill;
- 3.2 mm drill at implant length.

An implant was installed into the osteotomy with the coronal margin at the level of the cortical layer.

The final insertion torque was measured using the wrench provided by the implant company. At the end of the procedure, a cover screw was placed in each implant. Sutures were performed with Vicryl^®^ 4-0 (Ethicon^®^, Johnson & Johnson^®^, São José dos Campos, São Paulo, Brazil) for the periosteum and muscles and with nylon 4-0 (Ethicon^®^, Johnson & Johnson^®^, São José dos Campos, São Paulo, Brazil) for the skin. A bandage strip was placed over the wound for 3 days post-operatively.

### 2.8. Animal Maintenance

The rabbits were housed individually in cages (1 animal per 6000 cm^2^) at the Animal Facility of the Faculty of Dentistry, Ribeirão Preto, University of São Paulo. The facility maintained a controlled environment with air conditioning, exhaust fans providing 27 to 34 air changes per hour, and an automatic 12 h light–dark cycle. To protect the animals’ paws, a small plastic device was placed on the cage floors. They were provided with a specially formulated diet and had unlimited access to water.

Following the surgical procedure, the rabbits were temporarily transferred to smaller cages to limit their movement for 3 days, after which they were moved to standard-sized cages where they stayed until the end of the study. During the postoperative period and for the next three days, the animals were administered ketoprofen (3.0 mg/kg, every 12 h, intramuscularly; 10% Ketofen, Merial, Campinas, São Paulo, Brazil) and 2% tramadol hydrochloride (1.0 mg/kg, every 12 h, subcutaneously; Cronidor, Agener União Saúde Animal, Apucarana, Paraná, Brazil).

A strict monitoring protocol was followed throughout the study. This included daily assessments of basic biological functions, feeding and elimination habits, behavioral indicators of postoperative pain, and checks for any signs of infection, bleeding, or wound dehiscence.

### 2.9. Euthanasia

The animals were euthanized with an overdose (2.0 mL) of intravenous sodium thiopental (Thiopentax; Cristália, Itapira, São Paulo, Brazil) after 3 and 6 weeks. After experimental sites dissection, bone biopsies were removed and reduced to individual blocks, which remained in 10% paraformaldehyde for fixation.

### 2.10. Histological Preparation

The tibiae and femora were excised and preserved in 10% buffered formalin. Samples containing individual implants and adjacent hard tissue were sectioned. These samples underwent dehydration in a series of alcohol solutions, followed by embedding in resin (Technovit^®^ 7200 VLC, Kulzer, Wehrheim, Germany) and polymerization. Subsequently, each implant was cut along its longitudinal axis. Two ground sections were then prepared from each sample using slicing and grinding equipment (Exakt^®^, Apparatebau, Norderstedt, Germany), and, finally, were stained using Stevenel’s blue and alizarin red or toluidine blue.

### 2.11. Histological Examination

**Figure 3:**
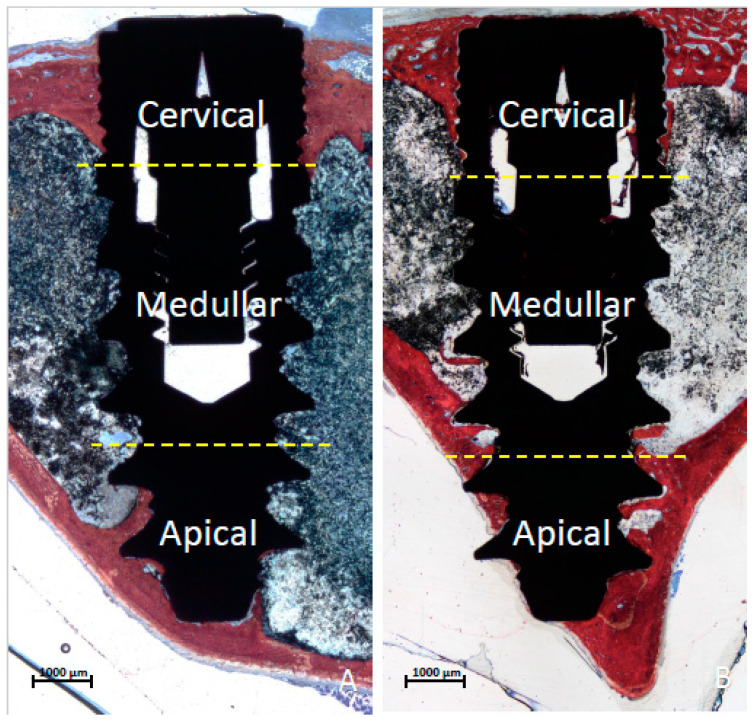
Photomicrographs of ground sections. (**A**) femur, drill site; (**B**) tibia, drill site. The three regions were delimited by lines, one passing above the first large thread and the other located above the second to last thread. The apical portion of the implant that extruded beyond the compact apical cortical layer was excluded from the analyses. Healing after 6 weeks, Stevenel’s blue and alizarin red stain.

Images were taken at about ×100 magnification. Histological evaluations were conducted using Image J software version 1.54 d (NIH, Bethesda, MD, USA). Marginal bone loss was evaluated from the implant margin and the area with the most coronal contact of the bone to the implant surface. Moreover, new bone and soft tissue in contact with the implant surface were evaluated in three regions, i.e., cervical, marrow, and apical. The three regions were delimited by lines [34], one passing above the first large thread and the other located above the second to last thread (Figure 3A,B). In some cases, the implant apex was extruding beyond the compact apical cortical layer. Consequently, the extruded part was excluded from the measurements.

### 2.12. Statistical Analyses

To assess the normality of the data distribution, the Shapiro–Wilk test was employed. For dependent variables, either the Paired *t*-test or the Wilcoxon test was utilized, whereas for independent variables, the Unpaired *t*-test or the Mann–Whitney test was applied. Statistical analyses were conducted using Prism 10.2.3 (GraphPad Software, LLC, San Diego, CA, USA). The primary variables were marginal bone resorption and BIC% in the cervical region. All the other variables were considered secondary variables.

## 3. Results

### 3.1. Clinical Outcomes

No clinical complications were observed, and all biopsies were available for histological processing so that *n* = 6 was obtained for each group. The final insertion torque in the femur was 25.0 ± 8.9 Ncm for the funnel technique and 29.6 ± 10.3 for the drill technique (*p* = 0.109). The final insertion torque in the tibia was 30.4 ± 14.6 Ncm for the funnel technique and 33.3 ± 13.1 Ncm for the drill technique (*p* = 0.504).

### 3.2. Descriptive Histological Evaluation

**Figure 4:**
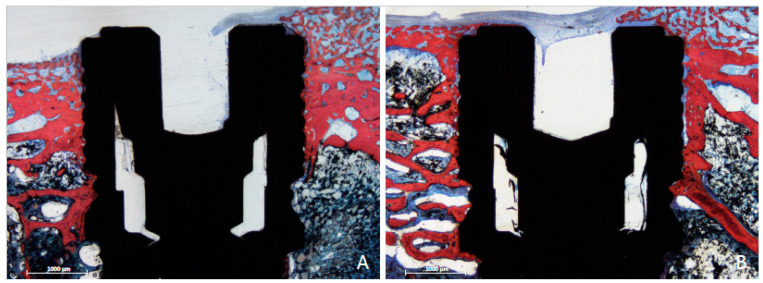
Photomicrographs of ground sections of the femur, 3 weeks of healing. In the cervical region, newly formed bone surrounded the implant collar as early as 3 weeks post-healing, often extending to cover the coronal margin of the implants. (**A**) Drill site. (**B**) Funnel site. Stevenel’s blue and alizarin red stain.

In the femur, the implants exhibited similar healing characteristics with both techniques. Spongy bone was enclosed within the cortical layers, which showed low bone density. However, in the cervical region, newly formed bone surrounded the implant collar as early as 3 weeks post-healing, often extending to cover the coronal margin of the implants (Figure 4A). Although the implant apex did not consistently reach the opposite cortical layer, newly formed bone bridges connected the apex to the cortical layer in both periods, even in the absence of direct contact (Figure 4B).

**Figure 5:**
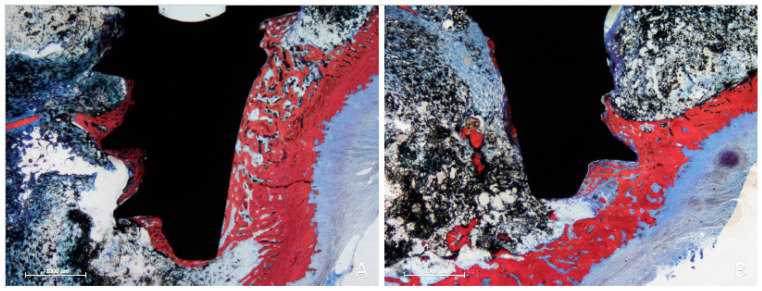
Photomicrographs of ground sections of the femur, 3 weeks of healing. Newly formed bone bridges connected the apex to the cortical layer, even in the absence of direct contact. (**A**) Drill site; (**B**) funnel site. Stevenel’s blue and alizarin red stain.

**Figure 6:**
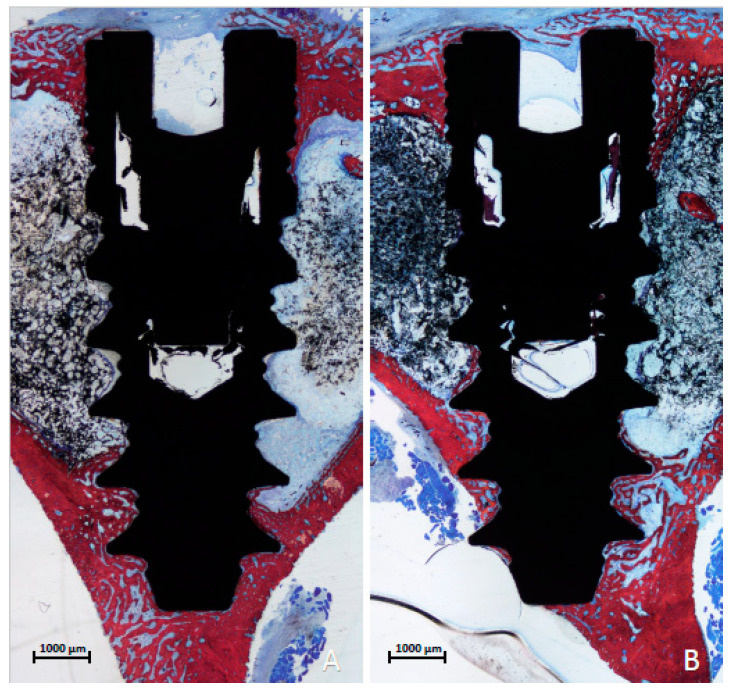
Photomicrographs of ground sections of the tibia region after 3 weeks of healing. New bone formation was evident in both cortical layers, establishing a robust connection with the implant surface in both the cervical and apical regions. No marginal bone loss was detected, and new bone formation was observed atop the cover screw. The cortical layers appeared dense, enclosing the bone marrow spaces, which lacked spongiosa ridges. (**A**) Drill site. (**B**) The implant apex penetrated (Figure 6A) and occasionally extended through the opposite cortical layer (Figure 6B). Stevenel’s blue and alizarin red stain.

In the tibia, a comparable healing response was observed with both techniques. New bone formation was evident in both cortical layers, establishing a robust connection with the implant surface in both the cervical and apical regions within just 3 weeks of healing. No marginal bone loss was detected, and new bone formation was observed atop the cover screw (Figure 5A,B). The cortical layers appeared dense, enclosing the bone marrow spaces, which lacked spongiosa ridges (Figure 5A,B). The implant apex penetrated (Figure 6A) and occasionally extended through the opposite cortical layer (Figure 6B).

### 3.3. Histological Assessments

Marginal bone resorption was not observed in any of the cases. However, in a very limited number of samples, histological analysis revealed the implant margin positioned slightly coronal to the cortical layer. This was attributed to the inclination of the implant relative to the coronal cortical plate, which was designed to ensure stability against the opposing cortical plates.

In the cervical region of both femur and tibia, similar BIC% (bone-to-implant contact percentages) were observed for funnel and drill sites after 3 weeks (Table 1) and 6 weeks of healing (Table 2). Differences, though not statistically significant, ranged between 2.8% and 4.7%, favoring one group or the other. However, higher BIC% values were observed in the femora compared to the tibiae in both periods.

BIC% in the cervical region of the femur remained stable between 3 and 6 weeks of healing, whereas in the tibia, particularly in the drill group, a reduction trend was noted, with a 12% decrease in BIC% (*p* = 0.041).

In the marrow spaces, small, non-significant differences in BIC% were noted at the 3-week mark between the funnel and drill sites. By 6 weeks, the BIC% values became very similar, with higher percentages in the femora than in the tibiae, although these differences were not statistically significant.

In the apical region of the femora, drill sites tended to have higher BIC% during both healing periods. No changes in BIC% were observed between the two periods of observation. In the tibiae, the BIC% increased between 3 and 6 weeks, particularly in the drill site (*p* > 0.01).

## 4. Discussion

This study aimed to assess the effects of two different drilling protocols on various bone densities. The two protocols showed no marginal bone resorption and comparable bone-to-implant contact (BIC) percentages in both the femur and tibia across all examined regions at both the 3-week and 6-week time points. However, some regional differences emerged when comparing BIC% in the femur and tibia. In the cervical region, the femur showed a higher BIC% than the tibia, particularly after 6 weeks. In contrast, the tibia exhibited higher BIC percentages in the apical region compared to the femur. Additionally, after 6 weeks, the marrow region of the femur presented a higher BIC percentage than the tibia, although this difference did not reach statistical significance.

Marginal bone resorption was not observed in any of the cases, confirming that both procedures for implant site preparation do not cause damage to the cervical cortical layer. However, in a very limited number of samples, histological analysis revealed the implant margin positioned slightly coronal to the cortical layer. This finding was likely due to the inclination of the implant relative to the coronal cortical plate, a strategy employed to achieve stability against the opposing cortical plates. These observations highlight the reliability of both techniques in preserving the integrity of the cervical cortical structure while ensuring implant stability. In a related rabbit study, blades of varying diameters were incorporated into the coronal segment of implants to prepare the cortical region of rabbit tibiae [35]. The experimental groups included blades with diameters differing from the implant collar by 0 μm, +50 μm, and +200 μm, with the latter two creating marginal gaps around the collar. This approach demonstrated effective osseointegration, with no marginal bone loss and complete closure of the gaps.

In a separate study, similar implants were tested in the alveolar bone crest of dogs [36]. Blade diameters differed from the implant collar as follows: a control group with −175 μm, causing compression of the marginal bone, and test groups with 0 μm, +50 μm, and +200 μm. Osseointegration at the buccal aspect was more coronally located in the test groups compared to the control, with the +50 μm and +200 μm groups showing a higher bone-to-implant contact percentage. These findings underscore the importance of avoiding compression on the marginal bone to facilitate better healing. Nonetheless, these variations did not reach statistical significance.

Also in the present study, no statistically significant differences were disclosed in BIC% between the two implant osteotomy preparations, suggesting that the funnel technique is a viable alternative. The use of an osteotome did not hinder implant integration and facilitated proper integration, even given the delicate bone structure of the rabbits’ tibia and femur.

A higher BIC percentage was also noted in the femur compared to the tibia, particularly in the 6-week period, which could be related to the differing initial bone quality in the regions of the tibia and femur. Despite the differences in marrow space preparation between the two techniques, no significant differences in BIC percentages were observed. However, the femur consistently showed a higher BIC percentage, likely due to its greater trabecular content. The lack of statistical significance is possibly due to high variability among the samples.

In the present study, three regions were evaluated: cervical, marrow, and apical. The analysis in the marrow region aimed to assess the potential influence of osteotome use on the bone marrow region. The effect was hypothesized to be more pronounced in the femur metaphysis, which has a higher spongiosa content. However, no positive effect of osteotome was observed at either the 3-week or 6-week healing periods.

These three regions have also been examined in other rabbit experiments [34,35]. In one study, implants with different surface characteristics were placed in the diaphysis and metaphysis of rabbit tibiae [34]. Healing was evaluated at four time points: 5, 8, 15, and 30 days. Interestingly, the implants placed in the diaphysis showed results consistent with those observed in the tibia of the present study, with high bone-to-implant contact (BIC%) in the cervical and apical regions and a low BIC% in the marrow region. Conversely, implants in the metaphysis demonstrated a high BIC% in the marrow region, likely due to the greater presence of spongiosa. In the current study, the femur exhibited a higher BIC% in the marrow region compared to the tibia, further highlighting the influence of spongiosa content on osseointegration.

The apical region demonstrated a strong healing response, achieving optimal integration with both techniques. In the tibia, higher BIC percentages were noted compared to the femur, particularly at the 6-week mark. This difference may be attributed to the tibia’s apex consistently reaching the opposite cortical layer, unlike in the femur, where such contact was rarely achieved. In the femur, new bone bridges often connected the apex to the cortical walls, allowing for integration but resulting in a lower percentage of new bone compared to the direct contact observed in the tibia. This pattern of bone formation has been noted in other studies, underscoring the importance of bicortical engagement for successful implant integration.

BIC percentages in the cervical region differed from those in the apical region for both the femur and tibia. After 6 weeks, the cervical region of the femur showed higher BIC values than the apical region. This finding could be due to the greater primary contact with the parent bone in the cervical region, which provided a more substantial source of new bone formation compared to the apex, where contact with the parent bone was infrequent. In contrast, the tibia exhibited higher BIC percentages at the apex compared to the cervical region. This difference can be explained by the triangular anatomy of the tibia’s apical region, which allows for more extended contact with the parent bone, fostering greater new bone formation and resulting in higher BIC percentages.

Both techniques provided good primary stability, with the drill technique showing a slight tendency toward higher torque values. This could be due to the smaller diameter of the osteotomy in the cervical region. Additionally, higher torque values were recorded in the tibia compared to the femur, likely because of the apex’s engagement with the opposite cortical layers in the tibia.

Two healing periods were selected based on data from a report that highlighted significantly faster bone-to-implant integration in rabbits compared to humans [37]. This article utilized data from other studies and introduced a concept termed the “interception” or “break-even point” of osseointegration. This point, represented graphically, marks where the proportional lines of old and new bone intersect, reflecting bone resorption and apposition rates on the implant surface. The timing for this intersection was approximately 4–6 days in rabbits, about twice as long in dogs, and four times as long in humans. A healing period of 3 to 6 weeks was deemed sufficient to capture complete healing in rabbits.

The limitations of this study are linked to the use of an animal model, the sites selected for implant installation, and the small sample size, a common characteristic of experimental research adhering to the 3Rs principles. Experimental studies often struggle to accurately mimic the clinical conditions encountered in human patients. Moreover, the highly controlled settings in which these experiments were performed may fail to reflect the multifaceted nature of real-world clinical situations, where patient-specific factors, underlying health conditions, and external influences significantly impact outcomes. Although experimental designs provide meticulous control over various parameters, they typically lack the extended observation periods required to evaluate the durability and long-term success of treatment results. An additional limitation is that the histologic evaluation relies solely on traditional staining methods. Immunohistochemistry could be more effective in detecting microscopic differences in bone turnover during the very early stages.

The results from the present study and those from the experiments that used blades incorporated in the implants [35,36] confirmed that a slight compression of the marginal bone or even a small marginal gap allow optimal marginal bone maintenance and osseointegration. However, clinical studies with at least one year follow-up analyzing the bone behavior should be organized to confirm or reject the result of the present study and clarify the effect of different implant site preparation techniques on peri-implant tissues in humans.

## 5. Conclusions

No marginal bone loss was observed in both techniques. No statistically significant differences in bone resorption or bone-to-implant contact around the implant collar were observed when comparing the two implant site preparation protocols across various bone densities. The use of osteotome did not influence the healing in the marrow region.

## Figures and Tables

**Table 1:** Three weeks of healing. Bone-to-implant contact evaluated at cervical, marrow, and apical levels in the femur and tibia bones. No statistically significant differences were found between funnel and drill techniques. # *p* < 0.05 between femur and tibia position.

	Femur		Tibia	
	Funnel	Drill	Funnel	Drill
Cervical	52.5 ± 10.2 ^#^	49.7 ± 14.7	39.9 ± 11.6 ^#^	43.7 ± 10.7
Marrow	6.9 ± 9.9	16.6 ± 13.3	8.8 ± 5.3	6.3 ± 6.4
Apical	34.9 ± 17.9	39.3 ± 12.4	51.8 ± 9.6	42.3 ± 7.3

**Table 2:** Six weeks of healing. Bone-to-implant contact evaluated at cervical, marrow, and apical levels in the femur and tibia bones. No statistically significant differences were found between funnel and drill techniques. # *p* < 0.05 between femur and tibia position.

	Femur		Tibia	
	Funnel	Drill	Funnel	Drill
Cervical	48.7 ± 10.8 ^#^	53.5 ± 10.7 ^#^	35.7 ± 9.0 ^#^	31.7 ± 7.2 ^#^
Marrow	14.5 ± 11.0	14.4 ± 14.8	6.7 ± 8.6	6.8 ± 9.6
Apical	33.6 ± 10.7 ^#^	38.7 ± 8.9 ^#^	57.7 ± 19.4 ^#^	60.9 ± 7.2 ^#^

## Data Availability

Data can be made available after request to the corresponding author.
